# Neural Coding of Natural Stimuli: Information at Sub-Millisecond Resolution

**DOI:** 10.1371/journal.pcbi.1000025

**Published:** 2008-03-07

**Authors:** Ilya Nemenman, Geoffrey D. Lewen, William Bialek, Rob R. de Ruyter van Steveninck

**Affiliations:** 1 Computer, Computational, and Statistical Sciences Division and Center for Nonlinear Studies, Los Alamos National Laboratory, Los Alamos, New Mexico, United States of America; 2 The Hun School of Princeton, Princeton, New Jersey, United States of America; 3 Joseph Henry Laboratories of Physics, Princeton University, Princeton, New Jersey, United States of America; 4 Lewis–Sigler Institute for Integrative Genomics, Princeton University, Princeton, New Jersey, United States of America; 5 Department of Physics, Indiana University, Bloomington, Indiana, United States of America; University College London, United Kingdom

## Abstract

Sensory information about the outside world is encoded by neurons in sequences of discrete, identical pulses termed action potentials or spikes. There is persistent controversy about the extent to which the precise timing of these spikes is relevant to the function of the brain. We revisit this issue, using the motion-sensitive neurons of the fly visual system as a test case. Our experimental methods allow us to deliver more nearly natural visual stimuli, comparable to those which flies encounter in free, acrobatic flight. New mathematical methods allow us to draw more reliable conclusions about the information content of neural responses even when the set of possible responses is very large. We find that significant amounts of visual information are represented by details of the spike train at millisecond and sub-millisecond precision, even though the sensory input has a correlation time of ∼55 ms; different patterns of spike timing represent distinct motion trajectories, and the absolute timing of spikes points to particular features of these trajectories with high precision. Finally, the efficiency of our entropy estimator makes it possible to uncover features of neural coding relevant for natural visual stimuli: first, the system's information transmission rate varies with natural fluctuations in light intensity, resulting from varying cloud cover, such that marginal increases in information rate thus occur even when the individual photoreceptors are counting on the order of one million photons per second. Secondly, we see that the system exploits the relatively slow dynamics of the stimulus to remove coding redundancy and so generate a more efficient neural code.

## Author Summary

Neurons communicate by means of stereotyped pulses, called action potentials or spikes, and a central issue in systems neuroscience is to understand this neural coding. Here we study how sensory information is encoded in sequences of spikes, using a combination of novel theoretical and experimental techniques. With motion detection in the blowfly as a model system, we perform experiments in an environment maximally similar to the natural one. We report a number of unexpected, striking observations about the structure of the neural code in this system: First, the timing of spikes is important with a precision roughly two orders of magnitude greater than the temporal dynamics of the stimulus. Second, the fly goes a long way to utilize the redundancy in the stimulus in order to optimize the neural code and encode more refined features than would be possible otherwise. This implies that the neural code, even in low-level vision, may be significantly context dependent.

## Introduction

Throughout the brain, information is represented by discrete electrical pulses termed action potentials or ‘spikes’ [1]. For decades there has been controversy about the extent to which the precise timing of these spikes is significant: Should we think of each spike arrival time as having meaning down to millisecond precision [2]–[5], or does the brain only keep track of the number of spikes occurring in much larger windows of time? Is precise timing relevant only in response to rapidly varying sensory stimuli, as in the auditory system [6], or can the brain construct specific patterns of spikes with a time resolution much smaller than the time scales of the sensory and motor signals that these patterns represent [3],[7]? Here we address these issues using the motion-sensitive neurons of the fly visual system as a model [8].

We bring together new experimental methods for delivering truly naturalistic visual inputs [9] and new mathematical methods that allow us to draw more reliable inferences about the information content of spike trains [10]–[12]. We find that as we improve our time resolution for the analysis of spike trains from 2 ms down to a fraction of a millisecond we reveal nearly 30% more information about the trajectory of visual motion. The natural stimuli used in our experiments have essentially no power above 30 Hz, so that the precision of spike timing is not a necessary correlate of the stimulus bandwidth; instead the different patterns of precise spike timing represent subtly different trajectories chosen out of the stimulus ensemble. Further, despite the long correlation times of the sensory stimulus, segments of the neural response separated by ∼30 ms provide essentially independent information, suggesting that the neural code in this system achieves decorrelation [13],[14] in the time domain, thereby enhancing the efficiency of the code on time scales relevant to behavior [15].

## Results

### Posing the problem

Flies exhibit a wide variety of visually guided behaviors, of which perhaps the best known is the optomotor response, in which visual motion drives a compensating torque, stabilizing straight flight [16]. This system offers many advantages for the exploration of neural coding and computation: There is a small group of identified, wide-field motion-sensitive neurons [8] that provide an obligatory link in the process [17], and it is possible to make very long, stable recordings from these neurons as well as to characterize in detail the signal and noise properties of the photoreceptors that provide the input data for the computation. In free flight, the trajectory of visual motion is determined largely by the fly's own motion through the world, and there is a large body of data on flight behavior under natural conditions [15], [18]–[20], offering us the opportunity to generate stimuli that approximate those experienced in nature. But the natural visual world of flies involves not only the enormous angular velocities associated with acrobatic flight; natural light intensities and the dynamic range of their variations are very large as well, and both of the fly's compound eyes are stimulated over more than 2π steradians. All of these features are difficult to replicate in the laboratory [21]. As an alternative, we have moved our experiments outside [9], so that flies experience the scenes from the region in which they were caught. We recorded from a single motion-sensitive cell, H1, while rotating the fly along trajectories modeled on published natural flight trajectories (see Methods for details). We should note that for technical reasons, these stimuli do not contain natural translation, pitch, and roll components, which may have an effect on the H1 responses; for other approaches to the delivery of naturalistic stimuli in this system see [22].

**Figure 1:**
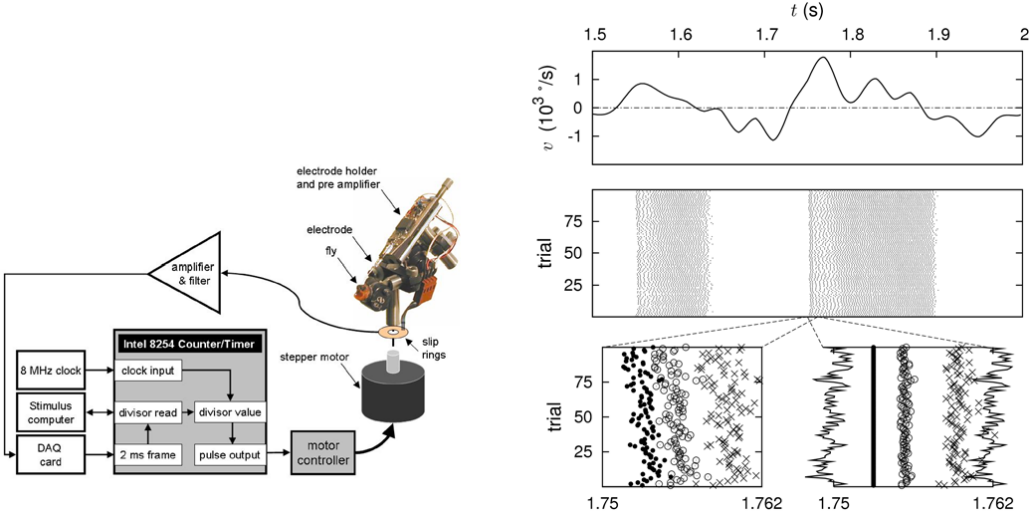
Neural responses to a natural stimulus ensemble. At left is a schematic of the experimental setup (see Methods for details). A fly was immobilized with wax, its body in a plastic tube, with its head protruding. Through a small hole in the back of the head an electrode was inserted to record extracellular potentials from H1, a wide field neuron sensitive to horizontal motion. This signal was amplified, fed through a slip ring system to a second stage amplifier and filter, and recorded by a data acquisition card. In synchrony with its master timer clock, the DAQ card generated a 500 Hz frame clock signal. Every 2 ms, through a bidirectional parallel port, this clock triggered a successive read of a divisor value from a file stored in the stimulus laptop computer. The Intel 8254 Counter/Timer chip used this divisor value to divide down the pulse frequency of a free running 8 MHz clock. In this way, in each successive 2 ms interval, and in strict synchrony with the data taking clock, a defined and evenly spaced burst of pulses was produced. These pulses drove the stepper motor, generating the angular velocity signal. A brief segment of this motion stimulus is shown in the top right panel, below which we plot a raster of action potentials from H1 in response to 100 repetitions of this stimulus. At bottom we expand the scale to illustrate (at left) that individual spikes following a transition from negative to positive velocity jitter from trial to trial by ∼1 ms: The standard deviations of spike times shown here are 0.72 ms for the first spike (•), 0.81 ms for the second spike (°), and 1.22 ms for the third spike (×). When we align the first spikes in this window, we see (at right) that the jitter of interspike intervals is even smaller, 0.21 ms for the first interval and 0.69 ms for the second interval. Our challenge is to quantify the information content of such precise responses.

A schematic of our experiment, and an example of the data we obtained, are shown in Figure 1. We see qualitatively that the responses to natural stimuli are very reproducible, and we can point to specific features of the stimulus—such as reversals of motion direction—that generate individual spikes and interspike intervals with better than millisecond precision. The challenge is to quantify these observations: Do precise and reproducible patterns of spikes occur just at some isolated moments, or does looking at the spike train with higher time resolution generally provide more information about the visual input?

Precise spike timing endows each neuron with a huge “vocabulary” of responses [1],[2], but this potential advantage in coding capacity creates challenges for experimental investigation. If we look with a time resolution of τ = 1 ms, then in each bin of size *τ* we can see either zero or one spike; across the behaviorally relevant time scale of 30 ms [15] the neural response thus can be described as a 30-bit binary word, and there are 2^30^, or roughly one billion such words. Although some of these responses never occur (because of refractoriness), and others are expected to occur only with low probability, it is clear that if precise timing is important then neurons can generate many more meaningfully distinguishable responses than the number that we can sample in realistic experiments.

### Progress in information estimation

Can we make progress on assessing the information content and meaning of neural responses even when we can't sample all of them? Recall that the information content is measured by the mutual information between the response and the stimulus that caused it [23]. This quantity measures (in bits) the reduction in the length of the description of the response spike train caused by knowing the associated velocity stimulus. Thus this mutual information is a difference of entropies [23] of the ensembles of all possible responses and the responses conditional on particular stimuli. Therefore, the problem of estimation of the information content of spike trains is essentially a problem of estimating the entropy of a probability distribution. This is known to be very hard when sampling is scarce, as in our problem [10],[24].

Some hope is provided by the classical problem of how many people need to be present in a room before there is a reasonable chance (about 50%) that at least two of them share a birthday. This number, which turns out to be *N*∼23, is vastly less than the number of possible birthdays, *K* = 365. Turning this argument around, if we didn't know the number of possible birthdays we could estimate it by polling *N* people and checking the frequency of birthday coincidences. Once *N* is large enough to generate several coincidences we can get a pretty good estimate of *K*, and, for *K*→∞, this happens when 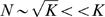. Some years ago Ma proposed that this coincidence counting method be used to estimate the entropy of physical systems from molecular dynamics or Monte Carlo simulations [25] (see also [26]). If these arguments could be generalized, it would become feasible to estimate the entropy and information content of neural responses even when experiments provide only a sparse sampling of these responses. The results of [10],[11] provide such a generalization.

**Figure 2:**
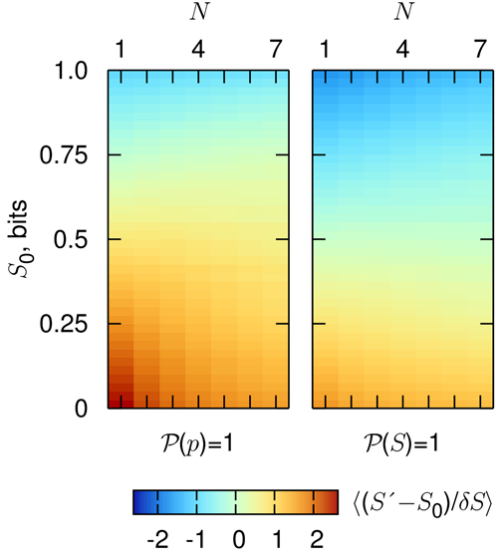
Systematic errors in entropy estimation. We consider a coin with unknown probability *p* of coming up heads; from *N* coin flips we try to estimate the entropy *S* = −*p*log_2_ *p*−(1−*p*)log_2_ (1−*p*); see Methods for details of the calculations. At left, we make Bayesian estimates starting from the prior hypothesis that all values of *p* are equally likely, P(*p*) = 1. We show how the best estimate *S*' differs from the true value *S*_0_ when this deviation is measured in units of the estimated error bar *δS* (posterior standard deviation); the color bar indicates the value of this scaled deviation. For small numbers of samples, the best estimate is systematically in error by more than two times the size of the error bar, so we would have false confidence in a wrong answer, even at intermediate values of the entropy, which are most relevant for real data. At right, we repeat the same procedure but with a prior hypothesis that all possible value of the entropy are equally likely, P(*S*) = 1. Systematic errors still appear, but they are more nearly compatible with the error bars, even at small *N*, and especially in the range of entropies, which is relevant to our experiments. Notice that here the distinction between the estimators extends to *N*∼*K* = 1; similarly, we expect the uniformization of P(*S*) to be advantageous when *N*<*K* even if *K*>>1.

To understand how the methods of [10] generate more accurate entropy estimates from small samples, it is useful to think about the simpler problem of flipping a coin under conditions where we don't know the probability *p* that it will come up heads. One strategy is to count the number of heads *n_H_* that we see after *N* flips, and identify *p* = *n_H_*/*N*; if we then use this “frequentist” or maximum likelihood estimate to compute the entropy of the underlying distribution, it is well known that we will underestimate the entropy systematically [24],[27],[28]. Alternatively, we could take a Bayesian approach and say that a priori all values of 0<*p*<1 are equally likely; the standard methods of Bayesian estimation then will generate a mean and an error bar for our estimate of the entropy given *N* observations. As shown in Figure 2, this procedure actually leads to a systematic *overestimate* of the entropy in cases where the real entropy is not near its maximal value. More seriously, this systematic error is larger than the error bars that emerge from the Bayesian analysis, so we would be falsely confident in the wrong answer.

Figure 2 also shows us that if we use a Bayesian approach with the a priori hypothesis that all values of the *entropy*, rather than *p*, are equally likely, then (and as far as we know, only then) we find estimates such that the systematic errors are comparable to or smaller than the error bars, even when we have seen only one sample. Thus the problem of systematic errors in entropy estimation is not, as one might have thought, the problem of not having seen all the possibilities; the problem rather is that seemingly natural and unbiased prior hypotheses about the nature of the underlying probabilities correspond to highly biased hypotheses about the entropy itself, and this problem gets much worse when we consider distributions over many alternatives. The strategy of [10] thus is to construct, at least approximately, a ‘flat prior’ on the entropy (see Methods for details). The results of [12] demonstrate that this procedure actually works for both simulated and real spike trains, where ‘works’ means that we generate estimates that agree with the true entropy within error bars even when the number of samples is much smaller than the number of possible responses. As expected from the discussion of the birthday problem, what is required for reliable estimation is that the number of coincidences be significantly larger than one [11].

We note that this estimation method is substantially different from other recent approaches, such as [4],[24],[29],[30], and we discuss the differences in some detail in the Discussion.

### Words, entropy and information

**Figure 3:**
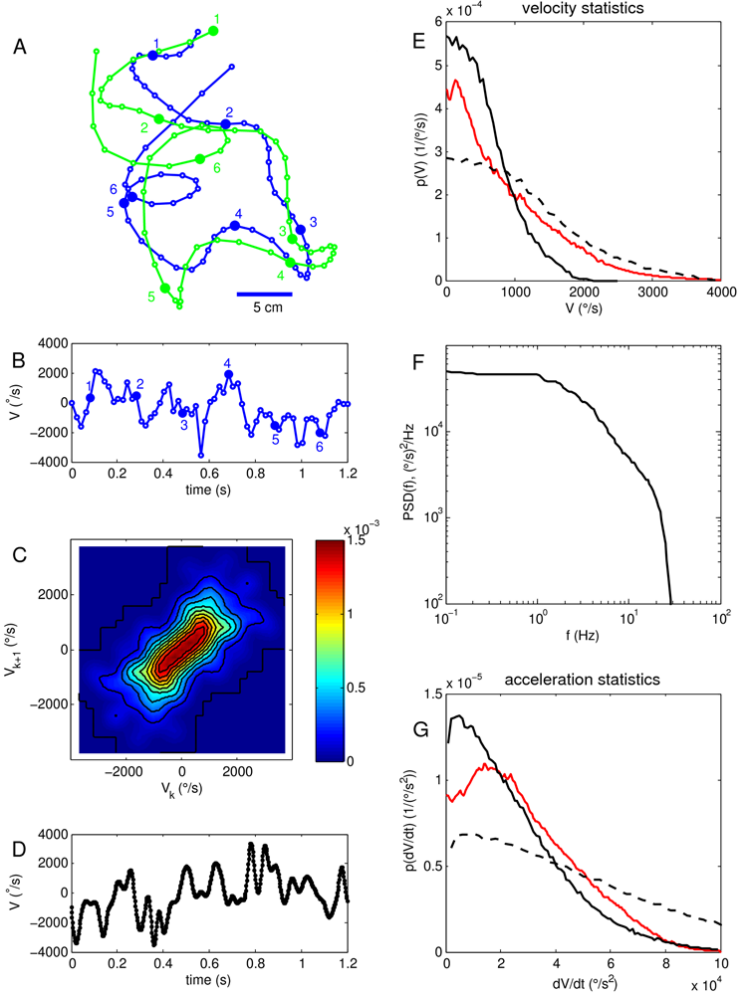
Constructing a naturalistic stimulus. (A) Digitized version of original video tracking data by Land and Collett [15]. The panel shows traces of a leading fly (blue) and a chasing fly (green). Successive points along the trajectories were recorded at 20 ms intervals. Every tenth point along each trajectory is indicated by a number. From these traces we estimated rotational velocities of the body axis by calculating the angular change in orientation of the trajectory from one point in the sequence to the next, and dividing by 20 ms. The result of this calculation for the leading fly is shown in panel (B). (C) >From these data (on both flies) we constructed a joint distribution, *P*(*V_k_*,*V_k_*_+1_), of successive velocities taken 20 ms apart. (D) Short sample of a trajectory constructed using the distribution in (C) as a Markov process, and then interpolating the velocity trace to 2 ms resolution. (E) Probability densities of angular velocity generated from this Markov process (black dashed line) and scaled down by a factor of two (black line) to avoid destabilizing the experiment; distributions are symmetric and we show only positive velocities. For comparison we show (red line) the distribution of angular velocities recorded for head motion of *Calliphora* during episodes of saccadic turning [20]. (F) Power spectrum of synthesized velocity signal, demonstrating the absence of power above 30 Hz. (G) As in (E) but for the accelerations. Note that the distribution of our synthesized and scaled signal was surprisingly close to that for saccadic head motions, as reported in [20].

**Figure 4:**
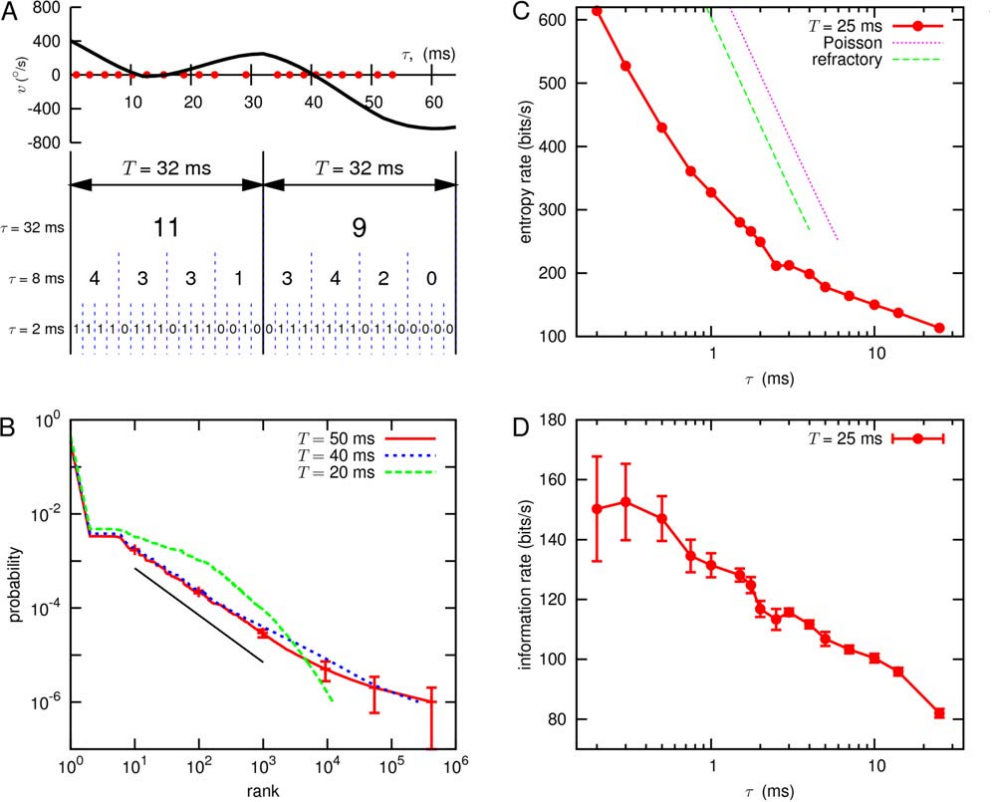
Words, entropy and information in the neural response to natural signals. (A) Schematic showing how we convert the sequence of action potentials into discrete ‘words’, that is, sequences of zeros and ones [31],[4]. As an example, at the top we show the stimulus and spike arrival times (red dots) in a 64 ms segment of the experiment. We may treat this as two successive segments of duration *T* = 32 ms, and divide these segments into bins of duration *τ* = 2, 8, or 32 ms. For sufficiently small *τ* (here, *τ* = 2 ms), each bin contains either zero or one spike, and so each neural response becomes a binary word with *T*/*τ* bits; larger values of τ generate larger alphabets, until at *τ* = *T* the response of the neuron is just the spike count in the window of duration *T*. Note that the words are shown here as non-overlapping; this is just for graphical convenience. (B) The distribution of words with *τ* = 1 ms, for various values of *T*; words are plotted in rank order. We see that, for large *T* (*T* = 40 or 50 ms) but not for small *T* (*T* = 20 ms), the distribution of words had a large segment in which the probability of a word is *P* ∝ 1/rank*^∝^*, corresponding to a straight line on this double logarithmic plot. Similar behavior is commonly observed for words in English, with *α* = 1, which we show for comparison (solid line); this is sometimes referred to as Zipf's law [48]. (C) The entropy of a *T* = 25 ms segment of the spike train, as a function of the time resolution *τ* with which we record the spikes. We plot this as an entropy rate, *S*(*T*,*τ*)/*T*, in bits/s; this value of *T* was chosen because this is the time scale on which visual motion drives motor behavior [15]. For comparison we show the theoretical results (valid at small *τ*) for a Poisson process [1], and a Poisson process with a refractory period [12], with spike rates and refractory periods matched to the data. Note that the real spike train has significantly less entropy than do these simple models. In [12] we showed that our estimation methods can recover the correct results for the refractory Poisson model using data sets comparable in size to the one analyzed here; thus our conclusion that real entropies are smaller cannot be the result of undersampling. Error bars are smaller than the data points. (d) The information content of *T* = 25 ms words, as a function of time resolution *τ*; again we plot this as a rate *R*_info_(*T*,*τ*) = *I*(*T*, *τ*)/*T*, in bits/s.

The tools described above allow us to estimate the entropy of neural responses. We first analyze a long experiment in which the fly experiences a continuous trajectory of motion with statistics modeled on those of natural flight trajectories (Figure 3; see Methods for details). As shown in Figure 4A, we examine segments of the response of duration *T*, and we break these segments into discrete bins with time resolution *τ*. For sufficiently small *τ*, each bin either has one or zero spikes, and hence the response becomes a binary word with *T*/*τ* bits, while in the opposite limit we let *τ* = *T*, and then the response is the total number of spikes in a window of size *T*; for intermediate values of *τ*, the responses are multi-letter words, but with larger than binary alphabet when more than one spike can occur within a single bin. An interesting feature of these words is that they occur with a probability distribution similar to the distribution of words in English (Zipf's law; Figure 4B). This Zipf-like behavior emerges only for *T>*20 ms, and was not observed in experiments with less natural, white noise stimuli [4].

With a fixed value of *T*, improving our time resolution (smaller *τ*) means that we distinguish more alternatives, increasing the “vocabulary” of the neuron. Mathematically this means that the entropy *S*(*T*,*τ*) of the neural responses is larger, corresponding to a potentially larger capacity for carrying information. This is shown quantitatively in Figure 4C, where we plot the entropy rate, *S*(*T*,*τ*)/*T*. The question of whether precise spike timing is important in the neural code is precisely the question of whether this capacity is used by the system to carry information [2],[4].

To estimate the information content of the neural responses, we followed the strategy of [4],[31]. The information content of the ‘words’ generated by the neuron is always less than the total size of the neural vocabulary because there is some randomness or noise in the association of words with sensory stimuli. To quantify this noise we choose a five second segment of the stimulus, and then repeat this stimulus 100 times. At each moment 0<*t*<5 s in the cycle of the repeated stimulus, we look across the one hundred trials to sample the different possible responses to the same input, and with the same mathematical methods as before, we use these samples to estimate the ‘noise entropy’ *S_n_*(*T*,*τ*|*t*) in this ‘slice’ of responses. The information which the responses carry about the stimulus then is given by *I*(*T*,*τ*)* = S*(*T*,*τ*)−〈*S_n_*(*T*,*τ*|*T*)〉*_t_*, where 〈…〉*_t_* denotes an average over time *t*, which implicitly is an average over stimuli. It is convenient to express this as an information rate *R*_info_ (*T*,*τ*) = *I*(*T*,*τ*)/*T*, and this is what we show in Figure 4D, with *T* = 25 ms, chosen to reflect the time scale of behavioral decisions [15].

The striking feature of Figure 4D is the growth of information rate with time resolution. We emphasize that this measurement is made under conditions comparable to those which the fly encounters in nature—outdoors, in natural light, moving along trajectories with statistics similar to those observed in free flight. Thus under these conditions, we conclude that the fly's visual system carries information about motion in the timing of spikes down to sub-millisecond resolution. Quantitatively, information rates double as we increase our time resolution from *τ* = 25 ms to below a millisecond, and the final ∼30% of this increase occurs between *τ* = 2 ms and *τ*≤0.5 ms. In the behaviorally relevant time windows [15], this 30% extra information corresponds to almost a full bit from this one cell, which would provide the fly with the ability to distinguish reliably among twice as many different motion trajectories.

### What do the words mean?

**Figure 5:**
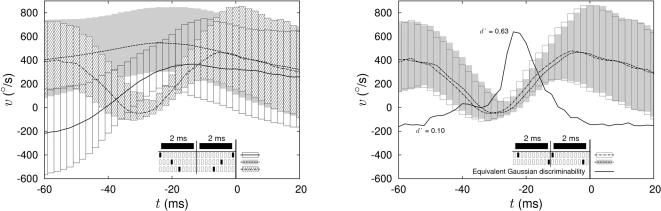
Fine spike timing differences and response conditional ensembles [32]. We consider five different neural responses, all of which are identical when viewed at *τ* = 2 ms resolution, corresponding to the binary pattern 11, spikes in two successive bins. At left, we consider responses which, at higher time resolution, correspond to different interspike intervals. At right, the interspike interval is fixed but higher time resolution revealed that the absolute spike arrival times differ. In each case, we compute the median motion trajectory conditional on the high time resolution response (lines) and we indicate the width of the distribution with bars that range plus and minus one quartile around the median. It is clear that changes in interspike interval encode changes in the distribution of stimulus waveform that are discriminable, since the mid-quartiles do not overlap. Changes in absolute timing are more subtle, and so we estimate the conditional distributions of velocity at each moment in time using the methods of [49], compute the overlap of these distributions, and convert the result into the equivalent signal-to-noise ratio *d'* for discrimination against Gaussian noise [50]; that is *d'* is a distance between the means of two unit variance Gaussians that have the same overlap as the distributions in question. Note that we compute this discriminability using single points in time; *d'* values based on extended segments of the waveforms would be even higher.

The information rate tells us *how much* we can learn about the sensory inputs by examining the neural response, but it doesn't tell us *what* we learn. In particular, we would like to make explicit the nature of the extra information that emerges as we increase our time resolution from *τ* = 2 ms to *τ*<1 ms. In other words, we should look at what additional features of the stimulus are encoded by finer spike timing. In the following we will present examples to highlight some of these features. We look at particular “words” in a segment of the neural response, as shown in Figure 5, and then examine the motion trajectories that corresponded to these words [32]. For simplicity, we consider all responses that had two spikes in successive 2 ms bins, that is the binary pattern 11 when seen at *τ* = 2 ms resolution. When we improve our time resolution to *τ* = 0.2 ms, some of these responses turn out to be of the form 10000000000000000001, while at the other extreme some of the responses have the two spikes essentially as close as possible given the refractory period, 00000100000000100000. Remarkably, as we sweep through these subtly different patterns—which all have the same average spike arrival time but different interspike intervals—the average velocity trajectory changes form qualitatively, from a smooth “on” (negative to positive velocity) transition, to a prolonged period of positive velocity, to a more complex waveform with off and on transitions in succession. Examining more closely the distribution of waveforms conditional on the different responses, we conclude that these differences among mean waveforms are in fact discriminable. Thus, variations in interspike interval on the millisecond or sub-millisecond scale represent significantly different stimulus trajectories.

A second axis along which we can study the nature of the extra information at high time resolution concerns the absolute timing of spikes. As an example, responses which at *τ* = 2 ms resolution are of the form 11 can be unpacked at *τ* = 0.2 ms resolution to give patterns ranging from 01000000001000000000 to 00000000010000000010, all with the same interspike interval but with different absolute arrival times. As shown in Figure 5, all of these responses code for motion trajectories with two zero crossings, but the times of these zero crossings shift as the spike arrival times shift. Thus, whereas the times between spikes represent the shape of the waveform, the absolute arrival time of the spikes marks, with some latency, the time at which a specific feature of the waveform occurs, in this case a zero crossing. Again we find that millisecond and sub-millisecond scale shifts generate discriminable differences.

**Figure 6:**
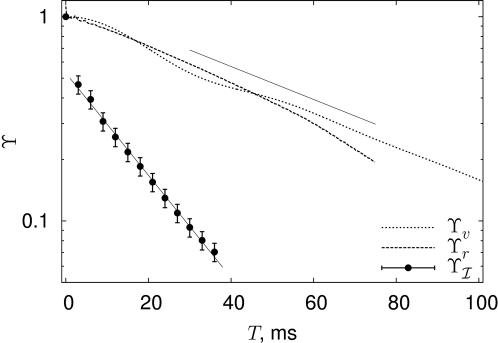
Redundancy reduction in the time domain. We measure the redundancy *Y_I_*(*T*,*τ*) (points with error bars) between words of length *T* in the neural response, as explained in the text. To allow exploration of large *T* we work at a time resolution *τ* = 3 ms. The redundancy is compared to correlations in the stimulus *Y_v_* = 〈*v*(*t*+*T*)*v*(*t*)〉/〈*v* ^2^〉 (dotted line) or correlations in the spike rate *Y_γ_* = 〈*δr*(*t*+*T*)*δr*(*t*)〉/〈*δr* ^2^〉 (dashed line). Note that the redundancy decays rapidly—we show an exponential fit with a time constant of 17.3 ms. In contrast, the correlations both in the stimulus and the firing rate decay much more slowly—the solid line, for comparison, shows an exponential decay with a time constant of 53.4 ms. Correlations in spike rate are calculated from a separate experiment on the same cell, with 200 repetitions of a 10 s stimulus drawn from the same distribution, that generated more accurate estimates of *r*(*t*).

The idea that sub-millisecond timing of action potentials can carry significant information is not new, but the clearest evidence comes from systems in which the dynamics of the stimulus itself has significant sub-millisecond structure, as in hearing and electroreception [6],[33]. For slow stimuli, the best recorded temporal precision is generally a few milliseconds, and is observed very early in the sensory processing [34]. Even for H1, experiments demonstrating the importance of spike timing at the ∼2 ms level [4],[35] could be criticized on the grounds that the stimuli had unnaturally rapid variations. It is thus important to emphasize that, in the experiments described here, H1 did not achieve millisecond precision simply because the input had a bandwidth of about a kiloHertz; in fact, the stimulus had a correlation time of ∼55 ms (Figure 6), and 99.9% of the stimulus power was contained below 30 Hz (Figure 3F). We are not aware of previous results where sub-millisecond temporal precision has been explicitly shown to encode such slow stimuli.

### Redundancy reduction

The long correlation time of these naturalistic stimuli also raises questions about redundancy—while each spike pattern considered in isolation may be highly informative, the long correlation time of the stimulus could very well mean that successive patterns carry information about essentially the same value of the instantaneous velocity. If so, that would mean that successive symbols are significantly redundant. Certainly on very short time scales this is true: Although *R*_info_(*T*,*τ*) actually increases at small *T* since larger segments of the response reveal more informative patterns of several spikes [35],[36], it does decrease at larger *T*, a clear sign of redundancy. However, this approach to a constant information rate is very fast: We measure the redundancy on time scale *T* by computing *Y_I_*(*T*,*τ*) = 2*I*(*T*,*τ*)/(2*T*,*τ*)−1, where *Y_I_* = 0 signifies that successive windows of size *T* provide completely independent information, and *Y_I_* = 1 that they are completely redundant. As shown in Figure 6, *Y_I_*(*T*,*τ*) decays rapidly, on a time scale of less than 20 ms. In contrast, correlations in the stimulus itself decay much more slowly, on the ∼55 ms time scale, and we find that the time dependent spike rate *r*(*t*) essentially has the same correlation time as the stimulus. The fact that coding redundancy decays three times more rapidly than the correlations of the time dependent firing rate indicates that the decorrelation of information is a process more intricate than simply filtering the stimulus. It suggests that there may be an adaptational mechanism at play that increases the overall efficiency of coding by exploiting the difference in time scales between stimulus changes and spike timing precision. If correct, this would imply that we should interpret neural firing patterns in context: The same pattern could signify slightly different stimulus depending on what went on before. This point merits further study, and may lead to further refinements in how we should interpret neural firing patterns, such as those shown in Figure 5. As far as we know this is the first direct information theoretic demonstration of temporal redundancy reduction in the context of neural coding.

### Bit rates and photon counting rates

The ability of the fly's visual system to mark features of the stimulus with millisecond precision, even at a ∼55 ms stimulus correlation time, was demonstrated in conditions where the visual input had very high signal-to-noise ratio. Previous work has suggested that this system can estimate motion with a precision close to the limits set by noise in the photoreceptors [37],[38], which is dominated by photon shot noise [39],[40]. The present experiments, however, were done under very different conditions: Velocities of motion were much larger, the fly's eye was stimulated over a much larger area, and light intensities outdoors were much larger than generated by laboratory displays. Light intensities in our experiment were estimated to correspond to up to about 1.1·10^6^ transduced photon/s per photoreceptor (see Methods). Is it possible that photon counting statistics are limiting the precision of H1, even at these high rates?

Because the experiments were done outdoors, there were small fluctuations in light intensity from trial to trial as clouds drifted by and obscured the sun. Although the range of these fluctuations was less than a factor two, the arrival times of individual spikes (e.g., the “first spike” after *t* = 1.75 s in Figure 1) had correlation coefficients of up to ρ = −0.42 with the light intensity, with the negative sign indicating that higher light intensities led to earlier spikes. One might see this effect as a failure of the system to adapt to the overall light intensity, but it also suggests that some of what we have called noise really represents a response to trial-by-trial variations in stimulus conditions. Indeed, a correlation between light intensity and spike time implies that the noise entropy *S_n_*(*T*,*τ*|*t*) in windows which contain these spikes has a significant contribution from stimulus variation, and should thus be smaller when this source of variation is absent.

More subtly, if photon shot noise is relevant, we expect that, on trials with higher light intensity, the neuron will actually convey more information about the trajectory of motion. We emphasize that this is a delicate question. To begin, the differences in light intensity were small, and we expect (at most) proportionately small effects. Further, as the light intensity increased, the total spike rate increased. Interestingly, this increased both the total entropy and the noise entropy. To see if the system used the more reliable signal at higher light intensities to convey more information, we have to determine which of these increases is larger.

To test the effects of light intensity on information transmission (see Methods for details), we divide the trials into halves based on the average light intensity over the trial, and we try to estimate the information rates in both halves; the two groups of trials differ by just 3% in their median light intensities. Since cutting the number of trials in half makes our sampling problems much worse, we focus on short segments of the response (*T* = 6 ms) at high time resolution (τ = 0.2 ms); note that these are still “words” with 30 letters. For this case we find that for the trials with higher light intensities the information about the motion stimulus is larger by Δ = 0.0204±0.0108 bits, which is small but significant at the 94% confidence level. We find differences with the same sign for all accessible combinations of *T* and *τ*, and the overall statistical significance of the difference thus is much larger. Note that since we were analyzing *T* = 6 ms windows, this difference correspond to Δ*R*∼3 bits/s, 1–2% of the total (cf. Figure 4). Thus even at rates of more than one million photons per second per receptor cell, small increases in photon flux produce proportionally small, yet measurable increases in the transmission of information about the motion stimulus.

## Discussion

We have found that under natural stimulus conditions the fly visual system generates spikes and interspike intervals with extraordinary temporal precision. As a consequence, the neural response carries a substantial amount of information that is available only at sub-millisecond time resolution. At this high resolution, absolute spike timing is informative about the time at which particular stimulus features occur, while different interspike intervals provide a rich representation of distinguishable stimulus features. These results clearly demonstrate that the visual system uses sub-millisecond timing to paint a more accurate picture of the natural sensory world, at least in this corner of the fly's brain. We emphasize again that here the sub-millisecond precision is not a result of an equally fast stimulus dynamics since the stimulus, in fact, has essentially no power at these frequencies. This is an important distinction, discussed in detail in [41]. In addition, an equally important observation is that the system performs efficiently both in the tasks of estimation and of coding, making use of the extra signal-to-noise provided by increased photon flux, even at daylight levels of light intensity. Perhaps of most interest, the analysis has made it possible to demonstrate a qualitative feature of the neural code in this system, namely the encoding of a temporally redundant stimulus in a neural signal of much shorter correlation time. At this point we can only speculate about the functional implications of this phenomenon, but at the very least it should give us pause in interpreting the code. Further study may reveal it to be an important feature of sensory coding and computation more generally, in particular under natural conditions where signals have high dynamic range, and show dramatic variations in reliability. We hope to be able to develop these ideas in more detail in the near future.

Finally, we note that our ability to reach these conclusions depends not just on new experimental methods that allow us to generate truly naturalistic stimuli [9], but critically on new mathematical methods that allow us to analyze neural responses quantitatively even when it was impossible for us to sample the distribution of responses exhaustively [10],[12]. The theoretical tools presented here were developed with the explicit aim of being efficient in estimating entropies in the severely undersampled regime. This is crucial in neurophysiological experiments, where large stable datasets are very difficult to obtain. Most previously described entropy estimation methods, such as [4], [24], [27]–[30],[42],[43], and others reviewed in [24], have relied on one of three different ways to overcome the undersampling problem. Some, for example [29], have chosen to define a metric on the space of responses, which makes it possible to “regularize” the problem by imposing similarity among probabilities of similar outcomes. Others, like [30], explore generative models for the data, which serves a similar regularizing function. Both approaches work well if and only if the underlying choices match the properties of the real data. The majority of recent approaches, such as [24], follow the third route and rely essentially on applying 1/*N* asymptotic corrections to the maximum likelihood estimator which means that they require mean bin occupancies *O*(1) to work. That leads to severe, and often impractical, demands on the size of the datasets as the cost of guaranteeing an estimator's performance. In contrast, the estimator presented here is based on counting coincidences, which still will occur even if the mean occupancy is much less than one. While we know that, in the worst case, even coincidence-based approaches may still require *O*(1) samples per possible outcome to produce low-bias and low-variance entropy estimates [44],[24], they may require substantially less data in simpler cases (in the best case scenario, to reach equal levels of resolution, the number of independent samples in the data set scales as the square-root of the number required by the other estimation methods. Or alternatively, with the same size dataset, the timing resolution is better by a factor of two.) For the data studied here, Nature cooperated: for example, to estimate noise entropies we use 100 samples for repeated stimuli for binary words of length 30 or more, so that the mean occupancy is <10^−7^. However, the success of the method could not have been predicted a priori, and the majority of our computational effort was spent not on calculation of information rates per se, but on answering the very delicate question of whether the NSB method can be trusted to have small bias for our data. This is why we caution the reader from using NSB as a simple black-box estimation tool, without checking if it really works first. Finally, we notice that our method for estimating entropies bears some resemblance to the work of Wolpert and Wolf [45], who used a single-beta Dirichlet prior to estimate functions of sparsely sampled probability distributions. A crucial distinction, however, is that instead of a single prior we use a *family* of Dirichlet priors to construct a prior *distribution* of entropies that is approximately flat (see Methods). We believe that, without a similar flattening of the distribution of entropies, any Bayesian method is bound to have large biases below bin occupancies of *O*(1).

Information theoretic approaches force us to formulate questions and quantify observations in unbiased ways. Thus, success in solving a problem in an information theoretic context leads to results of great generality. But success in an experimental context hinges on the solution of practical problems. We hope that the methods presented here contribute to solving an important practical problem, and will be a step toward wider application of information theoretic methods in neuroscience.

## Methods

### Neural recording and stimulus generation

H1 was recorded extracellularly by a short (12 mm shank length) tungsten electrode (FHC). The signal was preamplified by a differential bandpass instrumentation amplifier based on the INA111 integrated circuit (Burr-Brown). After amplification by a second stage samples were digitized at 10 kHz by an AD converter (National Instruments DAQCard-AI-16E-4, mounted in a Fieldworks FW5066P ruggedized laptop). In off line analysis, the analog signal was digitally filtered by a template derived from the average spike waveform. Spikes were then time stamped by interpolating threshold crossing times. The ultimate precision of this procedure was limited by the signal to noise ratio in the recording; for typical conditions this error was estimated to be 50–100 µs. Note that we analyzed spike trains down to a precision of τ = 200 µs, so that some saturation of information at this high time resolution may have actually resulted from instrumental limitations. The experiments were performed outside in a wooded environment, with the fly mounted on a stepper motor with vertical axis. The speed of the stepper motor was under computer control, and could be set at 2 ms intervals. The DAQ card generated a 500 Hz clock signal divided down from the same master clock that governs the AD sample rate. The stepper motor (SIG-Positec RDM566/50, 10,000 pulses per revolution, or 0.036°/pulse) was driven by a controller (SIG-Positec Divistep D331.1), which received pulses at a frequency divided down from a free running 8 MHz clock. Over the short time interval (*t*,*t*+2 ms) the stimulus velocity *v*(*t*) was determined by the pulse frequency, *f*(*t*), that the controller received. This in turn was set by the numerical value, *N_div_*(*t*), of a divisor: *f*(*t*) = 8MHZ/*N_div_*(*t*), and *v*(*t*) = (0.036) · *f*(*t*) °/s. Successive values of *N_div_*(*t*) were read every 2 ms from a stimulus file stored on a dedicated laptop computer. In this way, each 2 ms period the stepper motor speed was set to a value read from computer, keeping long-term synchrony with the data acquisition clock, with a maximum jitter of 1/(8 MHz) = 125 ns. The method for delivering pulses to the motor controller minimized the jerkiness of the motion by spacing the controller pulses evenly over each 2 ms interval. This proved to be crucial for maintaining stability of the electrophysiological recording.

### Controlling temperature

To stabilize temperature the setup was enclosed by a transparent plexiglass cylinder (radius 15 cm, height 28 cm), with a transparent plexiglass lid. The air temperature in the experimental enclosure was regulated by a Peltier element fitted with heat vanes and fans on the inside and outside for efficient heat dispersal, and driven by a custom built feedback controller. The temperature was measured by a standard J-type thermocouple, and could be regulated over a range from some five degrees below to fifteen degrees above ambient temperature. The controller stabilized temperature over this range to within about a degree. In the experiments described here, temperature was 23±1°C.

### Monitoring light intensity

A running overall measure of light intensity was obtained by monitoring the current of a photodiode (Hamamatsu S2386-44K) enclosed in a diffusing ping pong ball. After a current to voltage conversion stage, the photodiode signal was amplified by a logarithmic amplifier (Burr-Brown LOG100) operating over five decades. The probe was located ∼50 cm from the fly, and in the experiments the setup was always placed in the shade. The photodiode measurement was intended primarily to get a rough impression of relative light intensity fluctuations. To relate these measurements to outside light levels, at the start of each experiment a separate calibration measurement of zenith radiance was taken with a calibrated radiometer (International Light IL1400A using silicon detector SEL033/F/R, with radiance barrel). The radiance measurement was done over a limited spectral band defined by a transmission filter (International Light, WBS480) and an infrared absorption filter. In this way the radiometer's spectral sensitivity peaks close to the fly photoreceptor's 490 nm long wavelength maximum. However, it is about 20% broader than the fly's spectral sensitivity peak in the 350–600 nm range, and the photoreceptor's UV peak [46] was not included in this measurement. To relate this radiance measurement to fly physiology, the radiance reading was converted to an estimated effective fly photoreceptor photon rate, computed from the spectral sensitivity of the blowfly R1-6 type photoreceptor [46], the radiometer's spectral sensitivity and the spectral distribution of sky radiance [47]. The reading of the photodiode was roughly proportional to the zenith intensity reading, with a proportionality factor determined by the placement of the setup and the time of day. In the experiments, light intensities within the visual field of the fly ranged from about 2% to 100% of zenith intensity. To obtain a practical rule of thumb, the photodiode readings were converted to equivalent zenith photon flux values, using the current to zenith radiance conversion factor established at the beginning of the experiment. During the experiments the photodiode signal was sampled at 1 s intervals.

### Repeated stimuli

In their now classical experiments, Land and Collett measured the trajectories of flies in free flight [15]; in particular they reported the angular position (orientation) of the fly vs. time, from which we can compute the angular velocity *v*(*t*). The short segments of individual trajectories shown in the published data have a net drift in angle, so we include both the measured *v*(*t*) and −*v*(*t*) as parts of the stimulus. We used the trajectories for the two different flies in Figure 4 of [15], and grafted all four segments together, with some zero padding to avoid dramatic jumps in velocity, generating a 5 second long stimulus with zero drift, so that repetition of the angular velocity vs. time also repeated the angular position vs. time. Since Land and Collett reported data every 20 ms, we interpolated to generate a signal that drives the stepper motor at 2 ms resolution; interpolation was done using the MATLAB routine interp, which preserved the bandlimited nature of the original signal and hence did not distort the power spectrum.

### Nonrepeated stimulus

To analyze the full entropy of neural responses, it is useful to have a stimulus that is not repeated. We would like such a stimulus to match the statistical properties of natural stimulus segments described above. To do this, we estimated the probability distribution *P*[*v*(*t*+Δ*t*)|*v*(*t*)] from the published trajectories, where Δ*t* = 20 ms was the time resolution, and then used this as the transition matrix of a Markov process from which we could generate arbitrarily long samples; our nonrepeated experiment was based on a 990 s trajectory drawn in this way. The resulting velocity trajectories, in particular, had exactly the same distributions of velocity and acceleration as in the observed free flight trajectories. Although the real trajectories are not exactly Markovian, our Markovian approximation also captures other features of the natural signals, for example generating a similar number of velocity reversals per second. Again we interpolated these trajectories to obtain a stimulus at 2 ms resolution.

### Entropy estimation in a model problem

The problem in Figure 2 is that of a potentially biased coin. Heads appear with probability *p*, and the probability of observing *n* heads out of *N* flips is
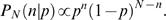
(1)


If we observe *n* and try to infer *p*, we use Bayes' rule [1] to construct
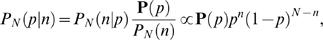
(2)where **P**(*p*) is our prior and 
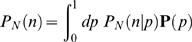
 is a normalization constant, which can be ignored. Given this posterior distribution of *p* we can calculate the distribution of the entropy,
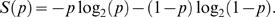
(3)


We proceed as usual to define a function *g*(*S*) that is the inverse of *S*(*p*), that is *g*(*S*(*p*)) = *p*; since *p* and 1-*p* give the same value of *S*, we choose 0<*g*≤0.5 and let *g* ˜ (*S*) = 1-*g*(*S*). Then we have
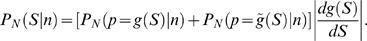
(4)


From this distribution, we can estimate a mean *S* ˜*_N_*(*n*) and a variance *σ*2(*n*,*N*) in the usual way. What interests us is the difference between *S* ˜*_N_*(*n*) and the true entropy *S*(*p*) associated with the actual value of *p* characterizing the coin; it makes sense to measure this difference in units of the standard deviation δ*S*(*n*,*N*). Thus we compute
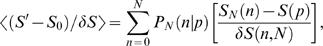
(5)and this is what is shown in Figure 2. We consider two cases. First, a flat prior on *p* itself, so that **P**(*p*) = 1. Second, a flat prior on the entropy, which corresponds to
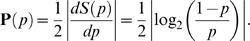
(6)


Here, 1/2 in front of the derivative accounts for two values of *p* being mapped into the same *S*. Note that this prior is (gently) diverging near the limits *p* = 0 and *p* = 1, but all the expectation values that we are interested in are finite.

### Entropy estimation: General features

Our discussion here follows [10],[12] very closely. Consider a set of possible neural responses labeled by *i* = 1,2,…,*K*. The probability distribution of these responses, which we don't know, is given by **p** ≡ {*p_i_*}. A well studied family of priors on this distribution is the Dirichlet prior, parameterized by β,
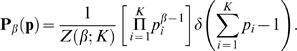
(7)


Maximum likelihood estimation, which identifies probabilities with frequencies of occurrence, is obtained in the limit *β* → 0, while *β* = 1 is the natural “uniform” prior. When *K* becomes large, almost any **p** chosen out of this distribution has an entropy 
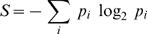
 very close to the mean value,
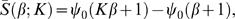
(8)where ψ_0_(x) = *d*log_2_ *Γ*(*x*)/*dx*, and *Γ*(*x*) is the gamma function. We therefore construct a prior that is approximately flat on the entropy itself by a continuous superposition of Dirichlet priors,
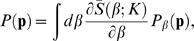
(9)and we then use this prior to perform standard Bayesian inference. In particular, if we observe each alternative *i* to occur *n_i_* times in our experiment, then
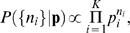
(10)and hence by Bayes' rule
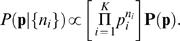
(11)


Once we normalize this distribution we can integrate over all **p** to give the mean and the variance of the entropy given our data {*n_i_*}. In fact, all the integrals can be done analytically except for the integral over β [10],[45]. Software implementation of this approach is available from http://nsb-entropy.sourceforge.net/. This basic strategy can be supplemented in cases where we have prior knowledge about the entropies. In particular, when we are trying to estimate entropy in “words” of increasing duration *T*, we know that *S*(T*,*τ*)≤*S*(*T*,*τ*)≤*S*(T*,*τ*)+*S*(*T*-T*,*τ*) for any T*<*T*, and thus it makes sense to constrain the priors at *T* using the results from smaller windows *T*', although this is not critical to our results. We obtain results at all integer values of *T*/*τ* for which our estimation procedure is stable (see below) and use cubic splines to interpolate to non-integer values as needed.

### Entropy estimation: Details for total entropy

There are two critical challenges to estimating the entropy of neural responses to natural signals. First, the overall distribution of (long) words has a Zipf-like structure (Figure 4B), which is troublesome for most estimation strategies and leads to biases dependent on sample size. Second, the long correlation times in the stimulus mean that successive words ‘spoken’ by the neuron are strongly correlated, and hence it is impossible to guarantee that we have independent samples, as assumed implicitly in Eq. (10). We tamed the long tails in the probability distribution by partitioning the space of responses, estimating entropies within each partition, and then using the additivity of the entropy to estimate the total. We investigated a variety of different partitions, including (a) no spikes vs. all other words, (b) no spikes, all words with one spike, all words with two spikes, etc., (c) no spikes, all words with frequencies of over 1000, and all other words. Further, for each partitioning, we followed [4] and evaluated *S*(*T*,*τ*) for data sets of different sizes *αN*, 0<*α*≤1. By choosing fractions of the data in different ways we separated the problems of correlation and sample size. That is, to check that our estimates were stable as a function of sample size, we chose contiguous segments of experiment, while to check for the impact of correlations we ‘diluted’ our sampling so that there were longer and longer intervals between words. Obviously there are limits to this exploration (one cannot access large, very dilute samples), but as far as we could explore the impact of correlations on our estimates was negligible once the samples sizes were sufficiently large. For the effects of sample size we looked for behavior of the form *S*(*α*) = *S*_∞_+*S*_1_/*α*+*S*_2_/*α* ^2^ and took *S*_∞_ as our estimate of *S*(*T,τ*), as in [4]. For all partitions in which the most common word (silence) was separated from the rest, these extrapolated estimates agreed and indicated negligible biases at all combinations of *τ* and *T* for which the 1/*α* ^2^ term was negligible (that is, did not change the extrapolation results by more than the extrapolation error) compared to the 1/*α*; this happened for all *τ*≥0.5 ms at *T*≤25 ms. For smaller *τ*, estimation failed at progressively smaller *T*, and to obtain an entropy rate for large *T* we extrapolated to *τ*/*T*→0 using
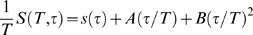
(12)where *s*(*τ*) was our best estimate of the entropy rate at resolution *τ*. All fits were of high quality, and the resulting error bars on the total entropy were negligible compared to those for the noise entropy. In principle, we could be missing features of the code which would appear *only* at high resolution for very long words, but this unlikely scenario is almost impossible to exclude by any means.

### Entropy estimation: Details for noise entropy

Putting error bars on the noise entropy averaged over time is more difficult because these should include a contribution from the fact that our finite sample over time is only an approximation to the true average over the underlying distribution of stimuli. Specifically, the entropies were very different in epochs that have net positive or negative velocities. We constructed the repeated stimulus, *v*(*t*) = −*v*(*t*+*T*_0_), with *T*_0_ = 2.5 s. As a result, the sum *S_n_*(*T*,*τ*|*t*)+*S_n_*(*T*,*τ*|*t*+*T*_1_) with *T*_1_≈*T*_0_ fluctuated much less as a function of *t* than the entropy in an individual slice. Because our stimulus had zero mean, every slice had a partner under this shift, and the small difference between *T*_0_ and *T*_1_ took account of the difference in latency between responses to positive and negative inputs. A plot of *S_n_*(*T*,*τ*|*t*)+*S_n_*(*T*,*τ*|*t*+*T*_1_) vs. time *t* had clear dips at times corresponding to zero crossings of the stimulus, and we partitioned the data at these points. We derived error bars on the mean noise entropy 〈*S_n_*(*T*,*τ*|*t*)*_t_*〉 by a bootstrap-like method, in which we constructed samples by randomly sampling with replacements from among these blocks, jittering the individual entropies *S_n_*(*T*,*τ*|*t*) by the errors that emerge from the Bayesian analysis of individual slices. These blocks are long enough to preserve temporal correlations within them, but correlations across the block boundaries are negligible in the original signal, validating the procedure. As with the total entropy, we extrapolated to otherwise inaccessible combinations of *T* and *τ*, now writing
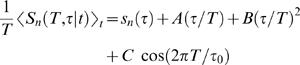
(13)and fitting by weighted regression. Note that results at different *T* but the same value of *τ* were strongly correlated, and so the computation of *χ* ^2^ was done using the full (non-diagonal) covariance matrix. The periodic term was important at small *τ*, where we could see structure as the window size *T* crossed integer multiples of the average interspike interval, *τ*_0_ = 2.53 ms. Error estimates emerged from the regression in the standard way, and all fits had *χ* ^2^∼1 per degree of freedom.

The procedures followed to get the total and noise entropy estimates in combination with the checks described above result in bias errors that are believed to be smaller than the random errors over the parameter range that we consider in all the analyses presented in this paper.

### Impact of photon flux on information rates

Since there were no responses to repeated and unrepeated stimuli recorded at exactly the same illuminations, we used the data from the repeated experiment to evaluate both the noise entropy and the total entropy. We were looking for minute effects, so we tightened our analysis by discarding the first two trials, which were significantly different from all the rest (presumably because adaptation was not complete), as well as excluding the epochs in which the stimulus was padded with zeroes. The remaining 98 trials were split into two groups of 49 trials each with the highest and the lowest ambient light levels. We then estimated the total entropy *S* ^(*h*,*l*)^(*T*,*τ*) for the high (*h*) and low (*l*) intensity groups of trials, and similarly for the noise entropy in each slice at time *t*, 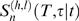. As above, assigning error bars was clearer once we formed quantities that were balanced across positive and negative velocities, and we did this directly for the difference in noise entropies,
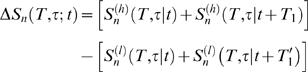
(14)where we allowed for a small difference in latencies 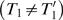 between the groups of trials at different intensities. We found that *ΔS_n_*(*T*,*τ*;*t*) had a unimodal distribution and a correlation time of ∼1.4 ms, which allowed for an easy evaluation of the estimation error.
